# Tentative Identification of Chemical Constituents in Liuwei Dihuang Pills Based on UPLC-Orbitrap-MS

**DOI:** 10.3390/metabo15080561

**Published:** 2025-08-21

**Authors:** Lanxiang Yang, Min Tao, Rongping Tao, Mingzhu Cao, Rui Wang

**Affiliations:** 1 School of Food and Bioengineering, Wuhu Vocational Technical University, Wuhu 241003, China; yanglanxiang@whit.edu.cn (L.Y.); 101151@whit.edu.cn (R.T.); 900148@whit.edu.cn (M.C.); 2 Gynaecology and Obstetrics, The Second Affiliated Hospital of Wannan Medical College, Wuhu 241003, China; 3 College of Pharmacy, Dali University, Dali 671003, China

**Keywords:** six-flavor Rehmannia Pill, ultra-high performance liquid chromatography-trap mass spectrometry, identification of chemical components, triterpenoids, cycloartane

## Abstract

Background: Liuwei Dihuang Pills, a classic traditional Chinese medicine formula, has been widely used in clinical practice for its multiple pharmacological effects. However, the systematic characterization and identification of its chemical constituents, especially the aqueous decoction, remain insufficient, which hinders in-depth research on its pharmacodynamic material basis. Thus, there is an urgent need for a comprehensive analysis of its chemical components using advanced analytical techniques. Methods: After screening chromatographic columns, the ACQUITY UPLC™ HSS T3 column (100 mm × 2.1 mm, 1.8 μm) was selected. The column temperature was set to 40 °C, and the mobile phase consisted of 0.1% formic acid in water (A) and 0.1% formic acid in acetonitrile (B). A gradient elution program was adopted, and the separation was completed within 20 min. Ultra-high performance liquid chromatography–Orbitrap mass spectrometry (UPLC-Orbitrap-MS) combined with a self-established information database was used for the analysis. Results: A total of 80 compounds were tentatively identified, including 13 monoterpenoids, 6 phenolic acids, 16 iridoids, 11 flavonoids, 25 triterpenoids, and 9 other types. Triterpenoids are mainly derived from Poria cocos and Alisma orientale; iridoids are mainly from Rehmannia glutinosa; monoterpenoids are mainly from Moutan Cortex; and flavonoids are mainly from Dioscorea opposita. Among them, monoterpenoids, iridoids, and triterpenoids are important pharmacodynamic components. The cleavage pathways of typical compounds (such as pachymic acid, catalpol, oxidized paeoniflorin, and puerarin) are clear, and their mass spectral fragment characteristics are consistent with the literature reports. Conclusions: Through UPLC-Orbitrap-MS technology and systematic optimization of conditions, this study significantly improved the coverage of chemical component identification in Liuwei Dihuang Pills, providing a comprehensive reference for the research on its pharmacodynamic substances. However, challenges remain in the identification of trace components and isomers. In the future, analytical methods will be further improved by combining technologies such as ion mobility mass spectrometry or multi-dimensional liquid chromatography.

## 1. Introduction

The prescription of Liuwei Dihuang Pills was first recorded in Straight Talk on Pediatric Medication Evidence [1]. In this formula, Rehmanniae Radix Praeparata nourishes yin and tonifies the kidney, fills essence, and enriches marrow, assisted by Alismatis Rhizoma to drain kidney dampness and reduce turbidity; Corni Fructus tonifies the liver and kidney, astringes, and consolidates, assisted by Moutan Cortex to drain liver fire; Dioscoreae Rhizoma invigorates the middle energizer and replenishes qi, and it also warms and tonifies the spleen and stomach, assisted by Poria to percolate dampness and invigorate the spleen. The entire formula combines three tonifying herbs and three draining herbs, exerting the effect of nourishing kidney yin through their collaborative actions [2,3,4]. At present, there are numerous studies on the quality differences in Liuwei Dihuang Pills. For example, Zhan Guoping et al. [5] analyzed the product quality of different manufacturers in accordance with the specified items in the Pharmacopoeia of the People’s Republic of China (abbreviated as Chinese Pharmacopoeia) 2015 edition and found that the samples from different manufacturers all met the basic requirements specified in the national standards, but there were significant differences in the contents of paeonol and loganin. Xia Yunqing et al. [6] combined fingerprint technology with the “component structure” theory to control and evaluate the quality of Liuwei Dihuang Pills and found that there were significant differences in the fingerprint similarity of samples from different batches produced by different manufacturers, as well as in the contents and quantity ratio relationships of the constituent elements of the Moutan Cortex component structure. Feng Xiaxia et al. [7] detected the contents of loganin, morroniside and paeonol in Liuwei Dihuang Pills and found obvious differences among samples from different manufacturers. Liuwei Dihuang Pills contain multiple medicinal ingredients and have complex chemical compositions, making it difficult to clarify their mechanism of action. Therefore, it is imperative to combine traditional Chinese medicine with modern technology to elucidate their mechanism of action. The material basis (chemical constituents) of traditional Chinese medicine determines its pharmacological activity and toxicity. Systematically elucidating the material connotations of traditional Chinese medicine is the foundation of quality control. To clarify the pharmacological and potential toxic components of Liuwei Dihuang Pills, it is highly necessary to carry out in-depth research on their material basis.

Liquid chromatography–mass spectrometry (LC-MS) technology has become a commonly used tool for the identification of multiple components in traditional Chinese medicine. Ultra-performance liquid chromatography–orbitrap mass spectrometry (UPLC-Orbitrap-MS), with advantages such as a wide range of selectivity, ultra-high sensitivity, and high-resolution mass measurement, has been used in recent years for the characterization and identification of complex traditional Chinese medicine systems [8].

In this study, the water-decocted liquid of Liuwei Dihuang Pills was used as the research object. Through systematic optimization of chromatographic and mass spectrometric conditions, a reversed-phase chromatography-based UPLC-Orbitrap-MS technique was established. By collecting data in both positive and negative ion modes, compared with the previous literature, more chemical constituents were identified from the water-decocted liquid of Liuwei Dihuang Pills in this research. Therefore, this study can lay a solid foundation for comprehensive research on the pharmacodynamic components and quality control of Liuwei Dihuang Pills.

## 2. Materials and Methods

### 2.1. Chemicals, Reagents and Materials

Rehmanniae Radix Praeparata (processed dried root tuber of Rehmannia glutinosa Libosch. from the genus Rehmannia in the family Scrophulariaceae), Corni Fructus (dried ripe sarcocarp of Cornus officinalis Sieb. et Zucc. from the genus Cornus in the family Cornaceae), Dioscoreae Rhizoma (dried rhizome of Dioscorea opposita Thunb. from the genus Dioscorea in the family Dioscoreaceae), Moutan Cortex (dried root bark of Paeonia suffruticosa Andr. from the genus Paeonia in the family Ranunculaceae), Poria (dried sclerotium of the fungus Poria cocos (Schw.) Wolf from the genus Poria in the family Polyporaceae), and Alismatis Rhizoma (tuber of Alisma orientale (Sam.) Juzep. from the genus Alisma in the family Alismataceae) were all purchased from Beijing Tongrentang Pharmacy. All the above medicinal materials were identified as genuine products by Professor Cao Kan from Wuhu Institute of Technology. Methanol (chromatographic grade, Dikma Corporation, Markham, ON, Canada); acetonitrile (chromatographic grade, Thermo Fisher Scientific, Waltham, MA, USA); and formic acid (chromatographic grade, Tedia Corporation, Fairfield, OH, USA) were also purchased. The UltiMate 3000 Ultra-High Performance Liquid Chromatograph (Thermo Fisher Scientific, Waltham, MA, USA); LTQ-Orbitrap Mass Spectrometer (Thermo Fisher Scientific, Waltham, MA, USA); and Milli-Q Pure Water System (Millipore Shanghai Trading Co., Ltd, Shanghai, China) were used. The reference substances, including pachymic acid (batch number: 21030401, purity 99.83%), polyporenic acid C (batch number: 190120, purity ≥ 98%), dehydrothymoic acid (batch number: 1911510, purity ≥ 98%), 16-oxo-alisol A (batch number: 230120, purity ≥ 98%), catalpol (batch number: 230120, purity ≥ 98%), Batatasin I (batch number: 232115, purity ≥ 98%), Loganin (batch number: 234113, purity ≥ 98%), 23-Acetyl alisol C (batch number: 133142, purity ≥ 98%), Paeonol(batch number: 432124, purity ≥ 98%), oxidized paeoniflorin (batch number: 332020, purity ≥ 98%) and puerarin (batch number: 470120, purity ≥ 98%), were all purchased from Shanghai Lvyuan Biotechnology Co., Ltd. (Shanghai, China).

### 2.2. Preparation of Liuwei Dihuang Pills Solution

A mixture of Rehmanniae Radix Praeparata, Corni Fructus, Dioscoreae Rhizoma, Alismatis Rhizoma, Moutan Cortex, and Poria was prepared at a ratio of 8:4:4:3:3:3 (by weight). The mixture was refluxed with a 10-fold volume of water for 2 h, and the filtrate was collected. The residue was further refluxed with an equal volume of water for 1 h, and the filtrates were combined. The combined filtrate was concentrated to 1 g·mL^−1^, stored at −20 °C until use. Prior to analysis, 0.5 mL of the concentrated solution was transferred to a 5 mL volumetric flask, diluted to volume with 70% methanol, and filtered through a 0.22 μm microporous membrane. The filtrate was used for subsequent analysis.

### 2.3. Chromatographic Conditions

Chromatographic separation was performed on an ACQUITY UPLC™ HSS T3 column (100 mm × 2.1 mm, 1.8 μm) maintained at 40 °C. The mobile phase consisted of 0.1% formic acid in water (A) and 0.1% formic acid in acetonitrile (B) with the following gradient elution program: 0–3.5 min: 15% B; 3.5–6 min: 15% B–30% B; 6–12 min: 30% B→70% B; 12–12.5 min: 70% B (isocratic)12.5–18 min: 70% B–100% B. The flow rate was set to 0.4 mL·min^−1^, and the injection volume was 5 μL.

### 2.4. Mass Spectrometric Conditions

Mass spectra were acquired in both positive and negative ion modes over a mass range of *m*/*z* 50–1500. The key parameters were as follows: spray voltage: +3.5 kV (positive mode), −3.2 kV (negative mode); ion transfer tube temperature: 320 °C; auxiliary gas heating temperature: 350 °C; collision energy: 20, 40, 60 eV (stepped); sheath gas pressure: 275,790 Pa (40 psi); auxiliary gas pressure: 137,895 Pa (20 psi); sweep gas pressure: 68,948 Pa (10 psi); RF lens amplitude (S-lens): 60; resolution: 70,000 FWHM (MS1), 17,500 FWHM (MS2); dynamic exclusion time: 5 s.

### 2.5. Data Processing

Data were processed using Thermo Scientific Xcalibur software (2.9.1) by comparing with multiple databases, including the Compound Discoverer database, Mass Frontier software 8.0, local high-resolution mass spectrometry database for traditional Chinese medicine components (OTC-ML), ChemSpider database, and mzCloud-Advanced mass spectrometry database. The analysis involved characterizing compounds based on retention time, accurate relative molecular mass, elemental composition, MS1 spectra (mass error ≤ 5 × 10^−6^), MS2 spectra (mass error ≤ 10 × 10^−6^), and fragmentation patterns. Chemical constituents were identified by matching with the component databases.

## 3. Results and Discussion

**Figure 1:**
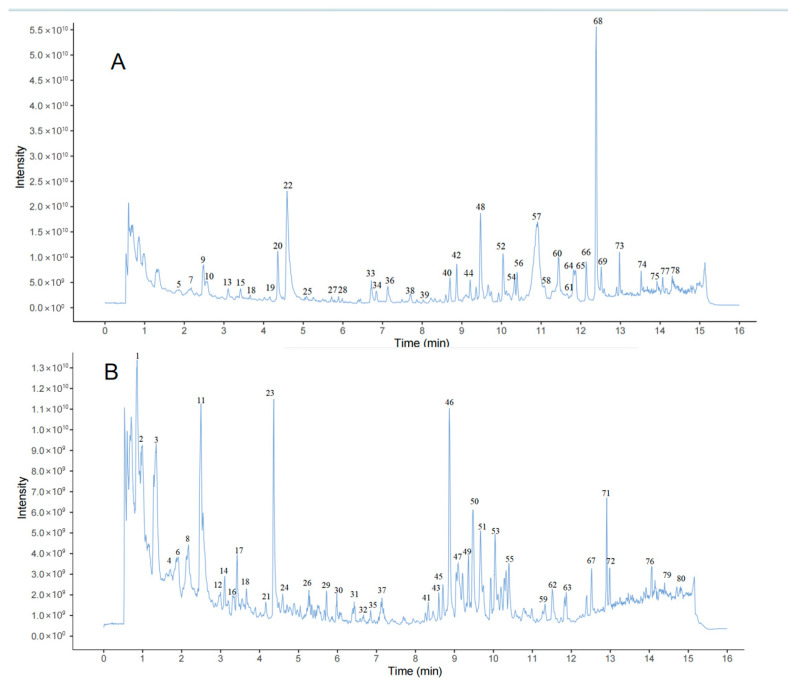
TIC chromatograms of Liuwei Dihuang Pills samples in positive and negative ion modes ((**A**) positive ion mode; (**B**) negative ion mode).

By means of reference substance comparison, self-established database retrieval, and literature verification, the high-resolution data of the water decoction of Liuwei Dihuang Pills collected under positive and negative ion modes were analyzed. A total of 80 compounds were identified or deduced, including 13 monoterpenoids, 6 phenolic acids, 16 iridoids, 11 flavonoids, 25 triterpenoids, and 9 other types. The literature reports indicate that monoterpenoids, iridoids, and triterpenoid components among them are important pharmacodynamic components [8,9]. The detailed information of the identification results is shown in Table 1, and the total ion current chromatograms of the water decoction of Liuwei Dihuang Pills under positive and negative ion modes are shown in Figure 1.

### 3.1. Structure Identification of Triterpenoids

**Figure 2:**
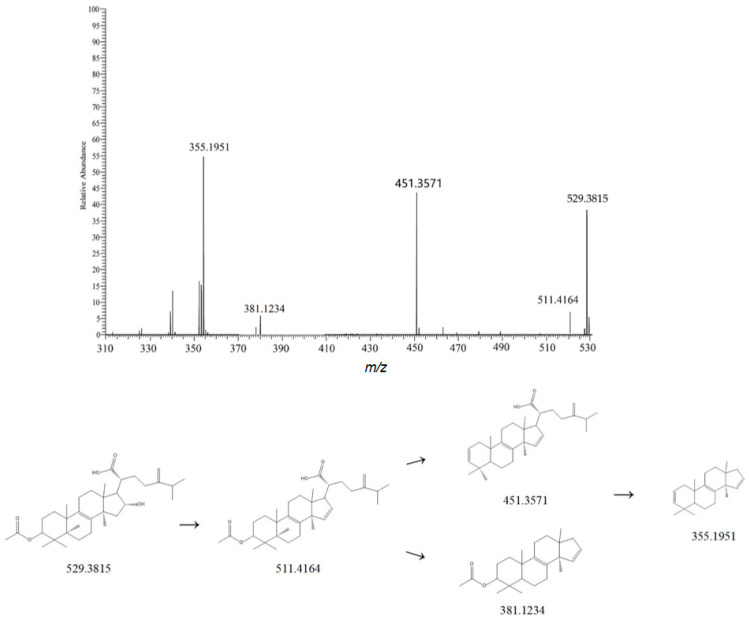
Possible mass spectrometry fragmentation pathways and secondary mass spectrum of Pachymic acid.

Twenty-five triterpenoid compounds were identified in Liuwei Dihuang Pills. They are mainly derived from Poria cocos and Alisma orientale and serve as the material basis for their potential efficacy. In Poria cocos, they mostly have a tetracyclic triterpenoid structure [20]. According to their structural types, they can be mainly divided into four categories as follows: lanost-8-ene type, lanost-7,9,(11)-diene type, 3,4-seco-lanost-8-ene type, and 3,4-seco-lanost-7,9,(11)-diene type. Pachymic acid belongs to the lanost-8-ene type in terms of structure [21]. Taking pachymic acid as an example, its molecular formula is C_33_H_52_O_5_. In the positive ion mode, its main ion peak is at *m*/*z* 529.3815. When the parent ion is bombarded, in one pathway, a molecule of H_2_O is first lost. Then, a molecule of CH_3_COOH and a molecule of C_9_H_16_O_2_ are successively lost, resulting in an ion peak at *m*/*z* 451.3571 [M+H-H_2_O-CH_3_COOH]^+^. In another pathway, a molecule of H_2_O is first lost, and then a molecule of C_9_H_16_O_2_ is lost successively, giving an ion peak at *m*/*z* 355.1951 [M+H-H_2_O-C_9_H_16_O_2_]^+^. By referring to the relevant literature and comparing the mass spectrometry information with the MzVault database, this compound was identified as pachymic acid [21]. Its fragmentation pattern is shown in Figure 2.

**Figure 3:**
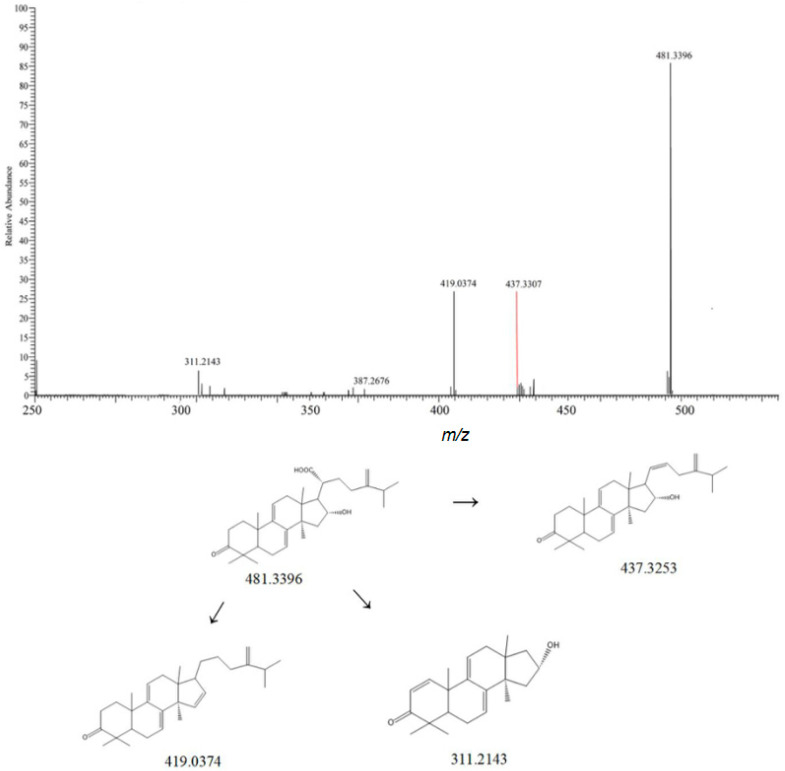
Possible mass spectrometric fragmentation pathways and secondary mass spectrum of Polyporenic acid C.

Compound **5** generates a quasi-molecular ion peak at *m*/*z* 481.3396 [M-H]^−^ in the negative ion mode. Its molecular weight is deduced to correspond to C_31_H_46_O_4_. Its secondary fragment ions include *m*/*z* 419.0374 [M-H-CO_2_-H_2_O]^−^, *m*/*z* 311.2143 [M-H-CH_4_-C_9_H_16_O_2_]^−^, and *m*/*z* 437.3253 [M-H-HCOOH]^−^. According to the mass spectrometry fragmentation rules in the positive and negative ion modes and by combining with references and comparison with reference substances, it is identified as polyporenic acid C [22]. Its mass spectrum and fragmentation rules are shown in Figure 3.

**Figure 4:**
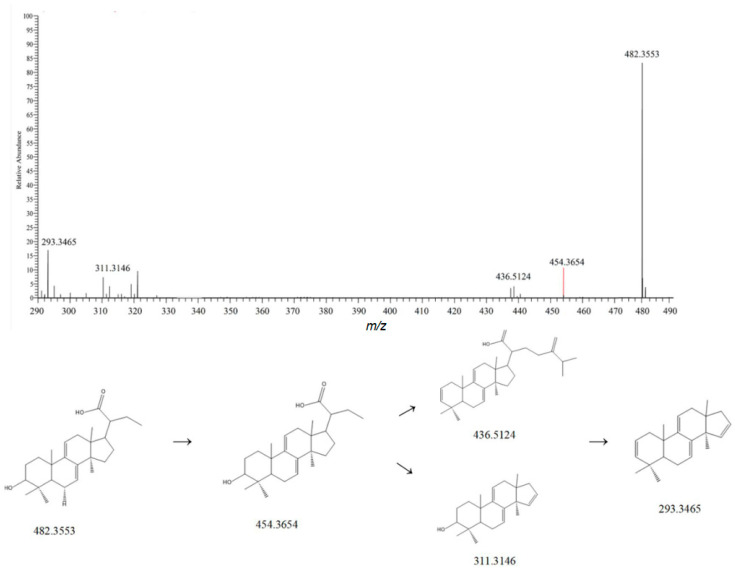
Possible mass spectrometric fragmentation pathways and secondary mass spectrum of dehydrothymoic acid.

Dehydrotumulosic acid belongs to the lanost-7,9,(11)-diene type in structure. Taking dehydrotumulosic acid as an example, its molecular formula is C_31_H_48_O4. In the positive ion mode, its main ion peak is at *m*/*z* 482.3553. When the parent ion is bombarded, in one pathway, it successively loses one molecule of H_2_O and then one molecule of C_9_H_16_O_2_, resulting in an ion peak at *m*/*z* 293.3465 [M-H-H_2_O-H_2_O-C_9_H_16_O_2_]^+^. In another pathway, it first loses one molecule of H_2_O and then one molecule of C_9_H_16_O_2_, giving an ion peak at *m*/*z* 311.3146 [M-H-H_2_O-C_9_H_16_O_2_]^+^. By referring to the relevant literature and comparing the mass spectrometry information with the MzVault database, this compound was identified as dehydrotumulosic acid [23]. Its fragmentation pattern is shown in Figure 4.

**Figure 5:**
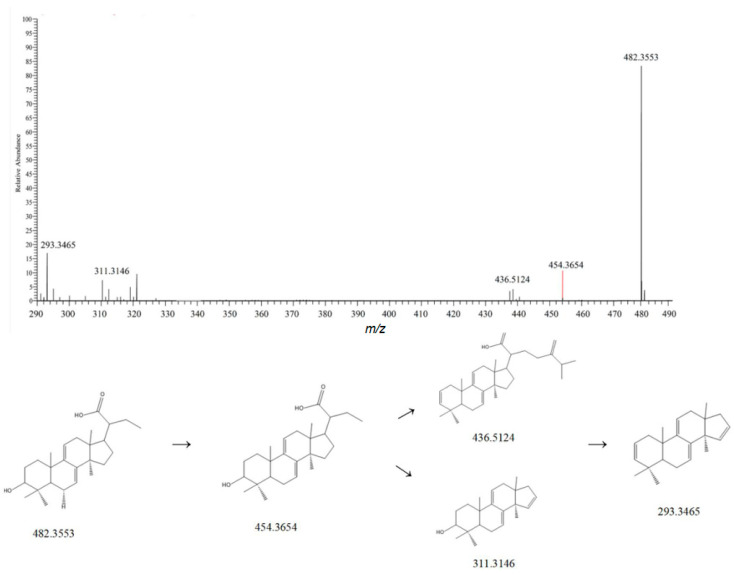
Possible mass spectrometric fragmentation pathways and secondary mass spectrum of 16-oxo-alisol A.

16-oxo-alisol A, alisol A, alisol C and alisol B are representative triterpenoid compounds of Alisma orientalis. Taking compound **47** as an example, a quasi-molecular ion peak of *m*/*z* 505.3510 [M+H]^+^ is generated in the positive ion mode, and its molecular weight is deduced to correspond to C_30_H_48_O_6_. Its secondary fragment ions include *m*/*z* 487.3418 [M+H-H_2_O]^+^, 469.3312 [M+H-2H_2_O]^+^, 415.2841 [M+H-C_4_H_10_O_2_]^+^, and 397.3101 [M+H-H_2_O-C_4_H_10_O_2_]^+^. Both the secondary fragments and the retention time are the same as those of the reference substance [24]. Therefore, the compound is identified as 16-oxo-alisol A, and the possible fragmentation pathway is shown in Figure 5.

### 3.2. Structural Identification of Iridoid Compounds

**Figure 6:**
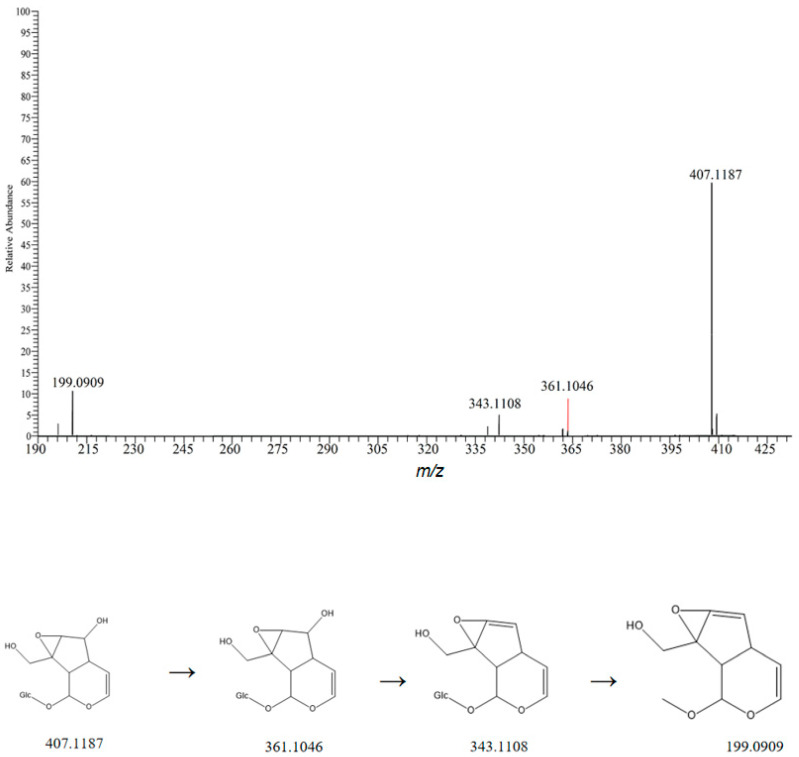
Possible mass spectrometric fragmentation pathways and secondary mass spectrum of catalpol.

Iridoid compounds mainly derive from Rehmanniae Radix, and their parent nucleus iridoid alcohols often combine with sugars to form glycosides. In negative ion mode, the fragmentation pathways of these compounds primarily involve glycosidic bond cleavage to lose glucose residues, cleavage of carboxyl and hydroxyl groups to lose neutral molecules such as CO_2_ and H_2_O, and loss of substituents on the parent nucleus. Compound **51** has a molecular formula of C_15_H_22_O_10_. In negative ion mode, it readily adds formic acid to show an adduct ion at *m*/*z* 407.1187 [M+COOH]^−^. Its secondary fragment ions mainly include the quasi-molecular ion peak at *m*/*z* 361.1046 [M-H]^−^, a fragment ion formed by further dehydration of this fragment at *m*/*z* 343.1108 [M-H-H_2_O]^−^ and a fragment ion formed by deglucosylation at *m*/*z* 199.0909 [M-H-Glc] [25]. These fragmentation characteristics are consistent with the literature reports, suggesting that the compound is catalpol. Its possible fragmentation pathway is shown in Figure 6.

### 3.3. Structure Identification of Monoterpenoids

**Figure 7:**
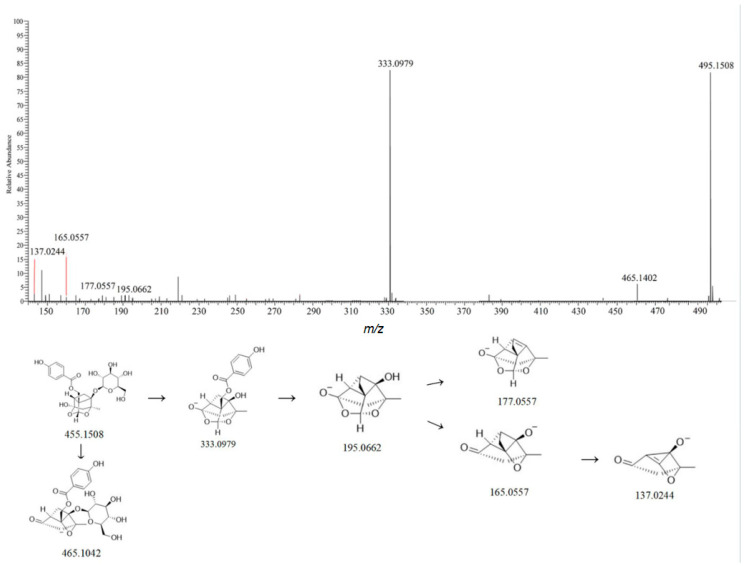
Possible mass spectrometric fragmentation pathways and secondary mass spectrum of Oxidized Paeoniflorin.

Cage-like pinane-type monoterpenoids are characteristic components of Moutan Cortex (Paeonia suffruticosa root bark) and its major bioactive constituents [26]. Mass spectrometry results show that monoterpenoids exhibit better mass spectral responses in negative ion mode, with quasi-molecular ion peaks mainly as [M-H]^−^ and [M+HCOO]^−^. Compound 39 showed a quasi-molecular ion peak at *m*/*z* 495.1519 [M-H]^−^ in negative ion mode. The assignment of fragment ions revealed that the parent ion lost neutral fragments CH_2_O and Glc to generate fragment ions *m*/*z* 465.1405 [M-H-CH_2_O]^−^ and 333.0980 [M-H-Glc]^−^. Further neutral losses of C_7_H_6_O_3_, H_2_O, CH_2_O, C_2_H_4_, or their combinations from these fragments produced ions 195.0654 [M-H-Glc-C_7_H_6_O_3_]^−^, 177.0555 [M-H-Glc-C_7_H_6_O_3_-H_2_O]^−^, 165.0556 [M-H-Glc-C_7_H_6_O_3_-CH_2_O]^−^, and 137.0248 [M-H-Glc-C_7_H_6_O_3_-CH_2_O-C_2_H_4_]^−^ [27,28,29]. The fragmentation pathway is shown in Figure 7, and the mass spectral data are consistent with the fragmentation rules of the compound. Therefore, it is speculated that this compound is oxidized paeoniflorin.

### 3.4. Structure Identification of Flavonoids

**Figure 8:**
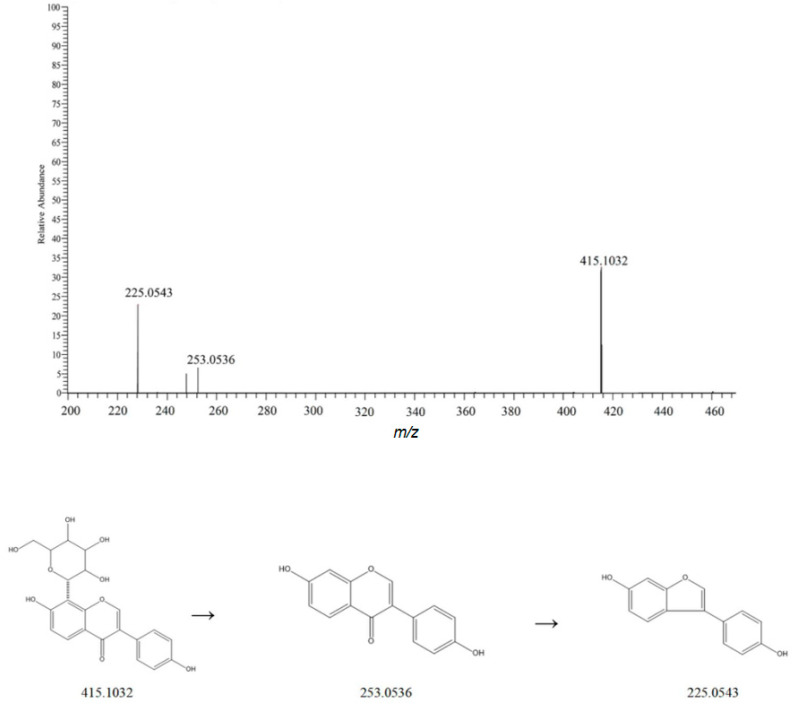
Possible mass spectrometric fragmentation pathways and secondary mass spectrum of puerarin.

Flavonoids are the main bioactive components of Chinese yam [30]. Mass spectrometry analysis showed that these flavonoids exhibited better mass spectral responses in the negative ion mode, and their quasi-molecular ion peaks were mainly [M-H]^−^. Taking compound 14 as an example, a quasi-molecular ion peak at *m*/*z* 415.1032 [M-H]^−^ was shown in the negative ion mode. By analyzing the fragment ions, it was found that the parent ion lost a neutral fragment Glc, generating a fragment ion at *m*/*z* 253.0506 [M-H-Glc]^−^. These fragments further underwent a neutral loss of CO, producing the following ion: 225.0543 [M-H-Glc-CO]^−^ [31,32,33]. Figure 8 shows this fragmentation pathway, and the mass spectrometry data are consistent with the fragmentation rules of the compound. Therefore, it is speculated that this compound is puerarin.

## 4. Discussion

This study systematically characterized and identified the chemical constituents in the water decoction of Liuwei Dihuang Pills using UPLC-Orbitrap-MS technology based on a self-established information database. Through optimization and screening of chromatographic columns with different silica cores and bonding groups (HSS T3, BEH C18, Zorbax Extend C18, Zorbax SB-C18, BEH Shield RP18, HSS C18 SB, Zorbax Eclipse Plus C18, Zorbax SB-Aq, CORTECS T3, HSS Cyano), it was found that the HSS T3 chromatographic column provided chromatographic peaks with good shape, numerous peaks, and high resolution. Subsequently, mobile phase systems including water–acetonitrile, 0.1% formic acid in water–acetonitrile, and 0.1% formic acid in water–0.1% formic acid in acetonitrile were compared. By comprehensively analyzing the resolution, peak shape, and response values of main components in chromatograms, 0.1% formic acid water–acetonitrile was selected as the mobile phase. Four different column temperatures (30, 35, 40, 45 °C) were compared, and it was determined that 35 °C offered good peak symmetry, optimal separation, high response values, and stable baseline. Representative compounds were selected for data collection using the Auto MS/MS positive ion scanning mode. With the average peak area from three injections as the evaluation index, optimization was performed on nozzle voltage (500, 1000, 1500, 2000 V), capillary voltage (2.0, 2.5, 3.0, 3.5, 4.0 kV), fragmentor (350, 360, 370,380, 390, 400 V), and collision energy (30, 35, 40, 45, 50 V). Considering the response values of all indicators, the optimized mass spectrometry parameters were determined as nozzle voltage 1000 V, capillary voltage 4.0 kV, fragmentor 380 V, and collision energy 40 V. Further optimization of the elution gradient achieved a good separation of chemical constituents in Liuwei Dihuang Pills within 20 min. Compared with previously reported studies on the chemical constituents of Liuwei Dihuang Pills [13,14], this research significantly improved the coverage of chemical constituent identification, providing a more comprehensive reference for studies on its active pharmaceutical substances. However, due to the complex and diverse chemical constituents of Liuwei Dihuang Pills, identifying trace components using UPLC-Orbitrap-MS technology remains challenging, and distinguishing numerous isomers solely based on secondary mass spectrometry information is difficult. In the future, enhanced characterization technologies based on ion mobility mass spectrometry or multi-dimensional liquid chromatography will be developed to more comprehensively and accurately characterize and identify trace chemical constituents in Liuwei Dihuang Pills, improving the reliability of structural identification and laying a more solid foundation for modern research.

## 5. Conclusions

By using UPLC-Orbitrap-MS for rapid qualitative identification and analysis of the chemical components in the extract of Liuwei Dihuang Pills, a total of 80 compounds were detected, including 13 monoterpenoids, 6 phenolic acids, 16 iridoids, 11 flavonoids, 25 triterpenoids, and 9 other types. This method is rapid, simple, and highly sensitive. It enriches the material basis of the efficacy of Liuwei Dihuang Pills and provides scientific data for subsequent quality research and pharmacological studies.

## Figures and Tables

**Table 1:** Identification of chemical components in Liuwei Dihuang Pills.

NO.	Rt	Measured Mass	Theoretical Mass	ppm	Frag Mention	Formula	Ion Mode	Compound	Classify	Crude Drugs	Reference
1	0.7	498.3345	497.8362	1.1	497.3254, 171.9834	C_31_H_46_O_5_	[M+H]^+^	6α-Hydroxyporia acid C	Triterpenoids	Poria cocos	[9]
2	0.99	472.3396	471.8673	2.1	469.3310, 448.4997	C_30_H_48_O_4_	[M+H]^+^	16α-Hydroxypsilocine	Triterpenoids	Poria cocos	[9]
3	1.33	454.3451	453.8907	0.8	423.2926, 371.2584	C_30_H_46_O_3_	[M-H]^−^	3-Dehydrotrametenolic acid	Triterpenoids	Poria cocos	[9]
4	1.72	486.3709	485.8845	−1.1	423.3619, 339.5969	C_31_H_50_O_4_	[M+H]^+^	Tumulosic acid	Triterpenoids	Poria cocos	[9]
5	1.81	481.3396	482.8573	−0.9	437.3307, 419.0374	C_31_H_46_O_4_	[M-H]^−^	Polyporenic acid C	Triterpenoids	Poria cocos	[9]
6	1.94	482.3553	483.8709	1.2	293.3465, 311.3146	C_31_H_48_O_4_	[M-H]^−^	Dehydrotumulosic acid	Triterpenoids	Poria cocos	[9]
7	2.18	514.7361	514.2213	−0.3	513.3565, 251.8673	C_33_H_50_O_6_	[M-H]^−^	3-O-Acetyl-16α-hydroxydehydrotrametenolic acid	Triterpenoids	Poria cocos	[9]
8	2.21	526.3658	525.8394	2.2	525.3566, 505.2605	C_33_H_50_O_5_	[M+H]^+^	Dehydropachymic acid	Triterpenoids	Poria cocos	[9]
9	2.42	529.3815	528.1691	−0.6	451.3571, 355.1951	C_33_H_52_O_5_	[M+H]^+^	Pachymic acid	Triterpenoids	Poria cocos	[9]
10	2.45	456.3604	455.9040	0.9	455.3517, 390.7080	C_30_H_48_O_3_	[M+H]^+^	Trametenolic acid	Triterpenoids	Poria cocos	[9]
11	2.62	287.055	287.0548	0.9	259.5467, 153.4792	C_15_H_10_O_6_	[M-H]^−^	Luteolin	Flavonoids	Dioscoreae Rhizoma	[10]
12	2.99	579.1708	579.1700	2.1	271.1449, 197.0597	C_15_H_10_O_5_	[M+H]^+^	Yuankanin	Flavonoids	Dioscoreae Rhizoma	[10]
14	3.10	415.1032	415.3687	1.9	253.0506, 225.0543	C_21_H_20_O_9_	[M-H]^−^	Puerarin	Flavonoids	Dioscoreae Rhizoma	[10]
13	3.11	470.3762	469.9056	−0.8	469.3673, 112.9855	C_31_H_50_O_3_	[M+H]^+^	Eburicoic acid	Triterpenoids	Dioscoreae Rhizoma	[10]
15	3.23	607.1668	607.1673	2.1	284.6423, 240.2348	C_28_H_32_O_15_	[M-H]^−^	Spinosin	Flavonoids	Dioscoreae Rhizoma	[11]
16	3.31	473.1089	473.1099	1.8	283.0606, 269.0459	C_23_H_22_O_11_	[M-H]^−^	6"-0-acetylgenistin	Flavonoids	Dioscoreae Rhizoma	[11]
17	3.45	299.0914	299.0922	0.6	445.1132, 299.0559	C_27_H_30_O_15_	[M-H]^−^	Mosloflavone	Flavonoids	Dioscoreae Rhizoma	[12]
18	3.65	271.0601	271.0601	1	153.0181, 135.0804	C_15_H_10_O_6_	[M+H]^+^	Apigenin	Flavonoids	Dioscoreae Rhizoma	[12]
19	3.71	415.1035	415.1032	2.1	253.0506, 225.0543	C_21_H_20_O_9_	[M-H]^−^	Puerarin	Flavonoids	Dioscoreae Rhizoma	[12]
24	4.65	148.1145	147.032 1	−2.4	147.5896, 129.1459	C_7_H_6_O_5_	[M+H]^+^	Malicacid4-Meester	Organic acids	fructus corni	[13]
25	4.75	345.0969	345.0997	0.6	271.0623, 240.7008	C_28_H_32_O_15_	[M-H]^−^	Eupatilin	Flavonoids	Dioscoreae Rhizoma	[11]
26	5.08	283.1464	284.30700	2.3	219.0919, 178.2154	C_17_H_16_O_4_	[M-H]^−^	Batatasin I	Flavonoids	Dioscoreae Rhizoma	[11]
27	5.28	164.1584	163.047 8	1.4	105.6283, 119.3586	C_8_H_8_O_3_	[M+H]^+^	ρ-coumaric acid	Phenolic acids	fructus corni	[14]
28	5.75	454.3471	453.8904	0.5	453.3358, 112.9850	C_30_H_46_O_3_	[M+H]^+^	Dehydrotrametenolic acid	Triterpenoids	fructus corni	[15]
29	5.82	568.4567	567.147 8	2.1	567.1986, 521.1786	C_25_H_38_O_16_	[M+H]^+^	Cormusglucoside F	Iridoid glycosides	fructus corni	[15]
30	5.91	635.2186	635.3114	1.3	465.5152, 300.1463	C27H14O18	[M+H]^+^	Trigalloylglucose	Phenolic acids	Moutan Cortex	[16]
31	5.95	139.0397	140.4301	1.4	136.1463, 121.2544;	C7H6O3	[M+H]^+^	p-hydroxybenzoic acid	Phenolic acids	Moutan Cortex	[16]
32	6.45	180.1574	179.034 0	−0.9	179.0304, 149.0081	C_9_H_8_O_4_	[M+H]^+^	Caffeic acid	Phenolic acids	fructus corni	[14]
33	6.62	391.4725	390.0291	2.1	341.5409, 221.1036	C_17_H_26_O_10_	[M+H]^+^	Loganin	Iridoid glycosides	fructus corni	[14]
34	6.75	388.4512	387.1425	−1.1	375.1286, 327.0721	C_19_H_30_O_9_	[M+H]^+^	Cornin	Iridoid glycosides	fructus corni	[14]
35	6.88	505.1558	510.2074	−3.1	205.0356, 167.0704	C_20_H_28_O_12_	[M+H]^+^	Paeonolide	Monoterpenoids	Moutan Cortex	[16]
36	6.91	488.1477	487.9792	−2	209.0472, 165.0523	C_15_H_20_O_8_	[M+H]^+^	Apiopaeonoside	Monoterpenoids	Moutan Cortex	[16]
37	7.15	388.3745	387.129 0	1.6	383.0439, 117.0354	C_19_H_30_O_9_	[M+H]^+^	Ketologanin	Monoterpenoids	fructus corni	[15]
38	7.18	523.1663	523.1663	0	323.0977, 199.0606	C_21_H_32_O_15_	[M+H]^+^	Rehmannioside A	Iridoid glycosides	Rehmanniae Radix	[17]
39	7.84	495.1519	495.1508	−2.3	281.0662, 195.0654	C_23_H_28_O_12_	[M-H]^−^	Oxypaeoniflorin	Monoterpals	Moutan Cortex	[16]
40	8.05	420.4125	419.1553	−1.2	373.1494, 358.1269	C_18_H_28O11_	[M-H]^−^	7-O-Methylmorroniside	Iridoid glycosides	fructus corni	[14]
41	8.34	404.3642	403.1236	−2.4	403.1240, 357.1191	C_17_H_24_O_11_	[M-H]^−^	Hastatoside	Iridoid glycosides	fructus corni	[14]
42	8.72	404.3662	403.1246	−2.4	225.0760, 179.0558	C_17_H_24_O_11_	[M-H]^−^	Secoxyloganin	Iridoid glycosides	fructus corni	[14]
43	8.77	358.3412	357.1186	1.27	195.0656, 173.0449	C_16_H_22_O_9_	[M-H]^−^	Sweroside	Iridoid glycosides	fructus corni	[14]
44	8.81	406.3824	405.1394	−1.9	373.1133, 243.0863	C_17_H_26_O_11_	[M-H]^−^	Morroniside	Iridoid glycosides	fructus corni	[14]
45	8.91	505.3531	505.4783	1.6	487.3445, 469.3329	C_30_H_48_O_6_	[M-H]^−^	Alismanol	Triterpenoids	Alismatis Rhizoma	[18]
46	8.93	507.3591	507.6980	1.3	453.3214, 397.2099	C_30_H_50_O_6_	[M-H]^−^	13,17-epoxyalisol A	Triterpenoids	Alismatis Rhizoma	[18]
47	9.15	505.3510	504.6950	−1.1	487.3408, 397.3415	C_30_H_48_O_6_	[M+H]^+^	16-oxo-alisol A	Triterpenoids	Alismatis Rhizoma	[18]
48	9.21	685.2207	685.2191	2.3	505.1564, 179.0556	C_27_H_42_O_20_	[M+H]^+^	Rehmannia D	Iridoid glycosides	Rehmanniae Radix	[17]
49	9.31	390.3475	389.1083	−1.3	389.1082, 345.1180	C_16_H_22_O_11_	[M-H]^−^	Secoxyloganic acid	Iridoid glycosides	fructus corni	[14]
50	9.45	361.1125	361.1135	−2.8	199.0603, 161.0441	C_15_H_22_O_10_	[M+H]^+^	Monomelittoside	Iridoid glycosides	Rehmanniae Radix	[17]
51	9.48	407.1187	407.7930	0.9	361.1046, 199.0909	C_30_H_46_O_6_	[M-H]^−^	Catalpol	Iridoid glycosides	Rehmanniae Radix	[17]
52	9.61	523.1661	523.1663	−0.4	463.1464, 343.1024	C_21_H_32_O_15_	[M+H]^+^	Melittoside	Iridoid glycosides	Rehmanniae Radix	[17]
53	10.11	510.5561	515.6617	−1.6	509.1615, 479.1123	C24H30O12	[M+H]^+^	Moudanpioside D	Monoterpals	Moutan Cortex	[16]
54	10.15	221.1895	222.3460	−2.2	203.1792, 161.1331	C_15_H_24_O	[M+H]^+^	Alismoxide	Triterpenoids	Alismatis Rhizoma	[18]
55	10.31	611.1036	617.2146	1.5	445.0933, 343.1069	C27H32O16	[M-H]^−^	Suffruticoside D	Monoterpals	Moutan Cortex	[16]
56	10.44	503.3362	503.5435	−0.9	485.3254, 467.3106	C_30_H_46_O_6_	[M+H]^+^	Dehydro-16-oxo-alisol A	Triterpenoids	Alismatis Rhizoma	[18]
57	10.45	471.368	471.9631	−0.9	471.3491, 453.3326	C_30_H_46_O_4_	[M-H]^−^	24-deacetylalisol O	Triterpenoids	Alismatis Rhizoma	[18]
58	10.95	515.6871	514.3679	−0.9	453.3388, 337.2803	C_32_H_50_O_5_	[M+H]^+^	23-acetyl alisol B	Triterpenoids	Alismatis Rhizoma	[18]
59	11.12	121.6761	122.8929	1.1	102.3516, 105.6456	C7H6O2	[M-H]^−^	Benzoic acid	Phenolic acids	Moutan Cortex	[16]
60	11.28	509.1879	509.1870	1.8	449.1672, 179.0555	C_21_H_34_O_14_	[M+H]^+^	Rehmannioside C	Iridoid glycosides	Rehmanniae Radix	[17]
61	11.43	347.1335	347.1342	−2	329.1227, 167.0704	C_15_H_24_O_9_	[M+H]^+^	Leonuride	Phenolic glycosides	Rehmanniae Radix	[17]
62	11.61	503.1623	508.1939	−2.5	463.1592, 179.0691	C_23_H_28_O_11_	[M-H]^−^	Peoniflorin	Triterpenoids	Moutan Cortex	[16]
63	11.65	461.1655	461.1659	−0.9	161.0431, 135.0435	C_20_H_30_O_12_	[M+H]^+^	decaffeoyl verbascoside	Phenolic glycosides	Rehmanniae Radix	[17]
64	11.81	487.3416	488.2031	−0.4	469.3312, 451.3205	C_30_H_46_O_5_	[M-H]^−^	Alisol C	Triterpenoids	Alismatis Rhizoma	[18]
65	11.88	631.5481	637.8636	−3.8	513.4863, 479.3541	C30H32O15	[M-H]^−^	Galonia paeoniflorin	Monoterpals	Moutan Cortex	[16]
66	11.93	939.2481	948.6406	−3.6	769.354, 617.6468	C41H32O26	[M-H]^−^	5-Acetylglucose	Monoterpals	Moutan Cortex	[16]
67	12.21	545.3466	545.3120	−1.2	485.3242, 467.3166	C_33_H_48_O_7_	[M-H]^−^	Alisol M 23-acetate	Triterpenoids	Alismatis Rhizoma	[18]
68	12.45	375.1281	375.1291	−2.7	213.0756, 169.0857	C_16_H_24_O_10_	[M+H]^+^	Loganic acid	Organic acids	Rehmanniae Radix	[17]
69	12.60	615.2791	621.4319	−0.1	431.6489, 281.5146	C30H32O14	[M+H]^+^	Moudanpioside H	Monoterpals	Moutan Cortex	[19]
70	12.63	600.3211	606.3243	−2.2	551.3947, 447.3923	C30H32O13	[M+H]^+^	Moudanpioside C	Monoterpals	Moutan Cortex	[19]
71	12.98	629.5461	635.8416	−1.7	599.3651, 507.6423	C31H34O14	[M+H]^+^	Moudanpioside J	Monoterpals	Moutan Cortex	[19]
72	13.01	487.3422	488.3174	0.9	469.3338, 451.3322	C_30_H_46_O_5_	[M-H]^−^	16-oxo-11-anhydro-alisol A	Triterpenoids	Alismatis Rhizoma	[18]
73	13.02	547.3626	547.6535	−0.6	529.3511, 415.2823	C_33_H_50_O_7_	[M+H]^+^	16-oxo-alisol A-23-acetate	Triterpenoids	Alismatis Rhizoma	[18]
74	13.53	791.2391	799.1514	0.8	623.1978, 593.18640	C_36_H_42_O_17_	[M+H]^+^	Paeoniflorin B	Monoterpals	Moutan Cortex	[19]
75	13.91	529.3519	528.6345	−0.8	511.3408, 469.3311	C_32_H_48_O_6_	[M+H]^+^	23-Acetyl alisol C	Triterpenoids	Alismatis Rhizoma	[18]
76	14.11	785.2512	785.2504	1	623.2196, 161.024	C_35_H_46_O_20_	[M+H]^+^	Purpureaside C	Cardiac glycosides	Rehmanniae Radix	[17]
77	14.15	390.3512	394.2547	1.2	327.1018, 151.0394	C16H24O8	[M+H]^+^	Moudanpioside G	Monoterpals	Moutan Cortex	[19]
78	14.31	315.0507	318.2012	−3.5	300.0239, 283.0768	C_16_H_12_O_7_	[M+H]^+^	Isorhamnetin	Flavonoids	Moutan Cortex	[19]
79	14.51	387.1294	387.1291	0.8	358.1248, 225.0764	C_17_H_24_O_10_	[M+H]^+^	Geniposide	Iridoid glycosides	Rehmanniae Radix	[17]
80	14.91	167.0326	166.7029	−0.3	152.0264, 122.0154	C_9_H_10_O_3_	[M+H]^+^	Paeonol	Phenolic acid	Moutan Cortex	[19]

## Data Availability

The data used to support the findings of this study are available from the corresponding author upon request.
